# Evaluating the role of vaccine to combat peste des petits ruminants outbreaks in endemic disease situation

**DOI:** 10.1186/s40781-014-0036-y

**Published:** 2015-01-20

**Authors:** Muhammad Abubakar, Shumaila Manzoor, Qurban Ali

**Affiliations:** National Veterinary Laboratory, Park Road, Islamabad, Pakistan; FAO FMD Project (GCP/PAK/123/USA), Islamabad, Pakistan

**Keywords:** Goat farming, PPR virus, Outbreaks, Treatment Regimes, Vaccination

## Abstract

Among the main intimidation to the sheep and goat population, PPR outbreaks are causing huge losses especially in endemic areas. During recent times, six outbreaks of PPR were confirmed at semi-organized goat farms/herds in various regions of Punjab province and Islamabad capital territory (ICT), Pakistan. The disease started after introduction of new animals at these farms with no history of previous PPR vaccination. The clinical signs appeared affecting respiratory and enteric systems and spread quickly. Disease caused mortality of 10-20% and morbidity of 20-40% within a time period of four weeks. Morbidity and mortality rates were 30.38% (86/283) and 15.55% (44/283), respectively. Three treatment regimes were executed to demonstrate the role of vaccination during outbreak at these farms. First was to use only the broad spectrum antibiotics (Penicillin & Streptomycin and/ or Trimethoprim and Sulfadiazine) at two farms (Texilla and Attock). Second treatment regime was to use the same broad spectrum antibiotic along with extensive fluid therapy (Farms at ICT-1 and ICT-2). The third regime was to use of broad spectrum antibiotic plus fluid therapy along with vaccinating the herd against PPR during first week of outbreak (ICT-3 and ICT-4). The third scheme of treatment gave the better results as there was no mortality in third week post-outbreak. Therefore, it is suggested to give proper importance to PPR vaccination along with conventional symptomatic treatment when dealing the PPR outbreaks in endemic disease conditions.

## Background

Pakistan at present is having more than 60 million head of goats, which consist of about 37 well- recognized breeds found in different regions of the country. Goats are important animals for humans for providing food in terms of milk and meat. They play main role in supporting millions of people who are poor and living in the rural areas. The main stock occurs in the form of nomadic and transhumant production system but the goat farming for commercial meat production is growing as a successful business.

PPR has caused significant economic loss in many parts of Africa and Asia that contain high densities of small ruminant population. The disease is characterized by sever pyrexia, anorexia, ulcerative necrotic stomatitis, diarrhea due to purulent oculo-nasal discharge and respiratory distress [1] which may be associated with coughing, foul offensive breath, pneumonia and death. Due to respiratory signs, the disease can be confused with contagious caprine pleuropneumonia (CCPP) and pasteurellosis. The disease was first time reported in Pakistan in 1991 [2].

Goats are the main victims of this disease but also involve sheep. The transmission of virus requires close contact between susceptible and infected animals in the febrile stage. The discharge from eyes, nose, mouth and the loose feces contain large amount of virus. Fine infected droplets are released into the air from these secretions and excretions particularly when infected animals cough and sneeze [3].

In Pakistan, during the last few years, PPR outbreaks have increased to an alarming level involving newer areas [4]. Keeping in view all the discussion, this report is designed to highlight the importance of various treatment strategies including vaccination while combating the PPR outbreaks in endemic disease situations to point out the possibilities to save goat farming from this risk.

## Methods

### Outbreaks description

**Figure 1:**
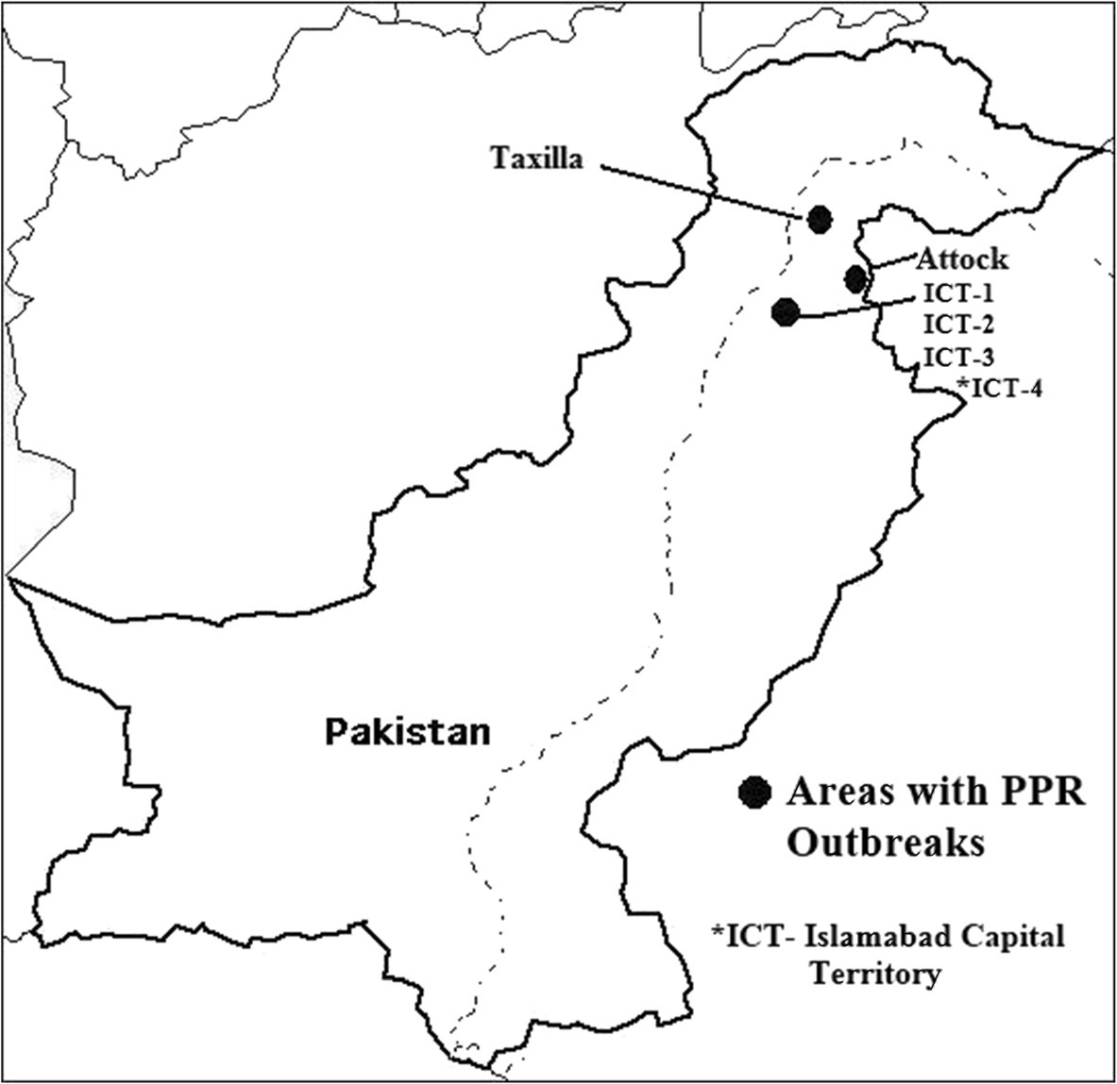
**Locations of farms/herds where PPR outbreaks were investigated.**

Outbreaks were investigated at six semi-organized commercial farms of goats in Islamabad Capital Territory (ICT) and two nearby areas of Punjab province (Attock, Taxilla) (Figure 1). The map of area is showing that the outbreaks occurred in close proximity. All the farms were having similar housing conditions with no history of previous PPR vaccination. The disease started after introduction of new animals to these farms. The animals (6-10 months of age) were purchased from different livestock markets with no history of PPRV vaccination.

### Study plan for treatment regimes

The study was planned to demonstrate the affects of various treatment strategies especially role of vaccination in the natural outbreak situations. There was three treatment regimes were selected and executed with each treatment regime was carried out at two farms.

First scheme was to use only broad spectrum antibiotics (Penicillin-Streptomycin and/or trimethoprim and sulfadiazine) at two farms (Texilla and Attock). The antibiotics were given intramuscularly daily at recommended dose.

Second treatment regime was to use the broad spectrum antibiotic along with extensive fluid therapy (Oral and intravenous) at two farms (ICT-1 and ICT-2). Some salt and sugar preparation (ORS) were added in drinking water while Ringer-lactate was given intravenously.

The third regime was to use of broad spectrum antibiotic plus fluid therapy as in second group along with vaccinating the herd against PPR (Pestivec^R^; Jordon) during first week of outbreak (ICT-3 and ICT-4). The vaccine was administered subcutaneously at dose rate of 1ml in adults and 0.5ml in young. Each treatment was started as soon as the disease was recognized at the farm.

### Collection of samples and laboratory confirmation

Nasal and ocular swabs were taken from sick animals while tissues from lungs, liver, spleen, intestines and lymph-nodes were collected from dead animals. Blood serum was also collected from sick animals.

Thirty swabs and sixty serum samples, collected from clinically suspected animals, were tested for PPRV confirmation by using Immuno-capture enzyme linked immune-sorbant assay (IcELISA) and Competitive ELISA (cELISA), respectively.

Representative samples of dead animals from each infected herds (4 from each herd) were also taken and tested through Ic ELISA and RT-PCR. In group-wise comparison, five swabs and ten serum samples were collected in each herd while all morbid animals were sampled for the confirmation of disease.

### Tests used for the confirmation of outbreaks

PPR antigen detection was performed using (Ic-ELISA) kit imported from World Reference Laboratory (Pirbright, UK). As recommended by the kit manufacturer, the final absorbance was measured at a wavelength of 492 nm [5].

The presence of viral nucleic acid from tissue samples was confirmed by Reverse Transcription-Polymerase Chain Reaction (RT-PCR). Total cellular RNA was extracted using the Qiagen-RNAeasy kit as per the manufacturer’s instructions. RT-PCR was performed for the F-gene of PPRV using one step RT-PCR kit (Invitrogen) as per the manufacturer’s instructions. PCR was carried out using PCR primers and conditions as described previously [6,7].

PPR antibodies were detected using cELISA kit (collectively produced by Biological Diagnostic Supplies Ltd, Flow Laboratories and The Pirbright Institute (formerly Institute for Animal Health). The OD values were converted to percentage inhibition and the samples with PI >50% were considered as positive [8].

## Results

Disease started with sudden onset of fever, respiratory and enteric clinical signs and spread quickly. At three farms, 86 out of 283 animals exhibited the clinical disease, giving morbidity rate of 30.38%. A total of 44 animals died with mortality rate of 15.55% (44/283) (Table 1).

**Table 1:** **Farm-wise distribution of PPR outbreaks with specie and mortality rate**

**Area/Farm**	**Animal species**	**Total animal kept**	**Diseased**	**Mortality**	**Morbidity rate %**	**Mortality rate %**
**Taxilla**	Goat	55	17	10	30.91	18.18
**Fateh-Jung (Attock)**	Goat	67	16	09	23.88	13.43
*ICT-1	Goat	34	11	04	32.35	11.76
**ICT-2**	Goat	42	16	07	38.09	16.67
**ICT-3**	Goat	57	15	09	26.32	13.43
**ICT-4**	Goat	28	11	05	39.28	17.86
		283	86	44	30.38	15.55

*Islamabad Capital Territory.

Among the samples tested, swab and tissue samples were positive with both Ic-ELISA and RT-PCR while all the serum samples were found positive for PPR antibodies (Table 2).

**Table 2:** **Summary of samples tested with different tests**

**Type of samples**	**Samples tested**	**Test Applied & Samples Positive**			
		**cELISA**	**IcELISA**	**RT-PCR**	**Results**
Swab (ocular and nasal)	30	--	21	25	Positive for PPR antigen + F-gene detection
Serum	60	52	--	--	Positive for PPR antibodies
Tissue (Lung, Spleen and Lymph-nodes)	24	--	19	22	Positive for PPR antigen + F-gene detection

There was a variation of mortality pattern in groups under different treatment regimes. In group I, the mortality continued up-to fourth week of clinical outbreak while the clinical disease sustained up to sixth week of its onset. In group II, the mortality stopped in third week and still the clinical disease continued up to fourth week post clinical disease.

In group-III, the mortality stopped in second week while the clinical disease continued up to fourth week but its symptoms were mild (Table 3). Mortality rate on first week of the outbreak was similarly high in all groups but it dropped appreciably in second week in group 2 and 3. It further dropped in group- 2 in third week but there was no mortality in group 3 while the mortality continued in group-1 till fourth week.

**Table 3:** **Treatment-wise distribution of mortality in selected PPR outbreaks**

**Treatment group**	**Area/farm**	**Overall mortality**	**Mortality in first week**	**Mortality in second week**	**Mortality in third week**	**Mortality in fourth week**
	**Taxilla**	10	4	3	2	1
**Group-1**	**Fateh-Jung (Attock)**	09	5	2	1	1
**Group-2**	**ICT-1**	04	2	1	1	0
	**ICT-2**	07	4	2	1	0
**Group-3**	**ICT-3**	09	6	3	0	0
	**ICT-4**	05	4	1	0	0
		**44**	**25**	**12**	**5**	**2**

## Discussion

The study presents an important scenario for PPR disease and its treatment in endemic disease situation in Pakistan. Due to lack of awareness of disease and no organized vaccination program, the disease has become endemic in Pakistan [9-11]. In the past PPR outbreaks were diagnosed only on the basis of clinical signs because of laborious laboratory procedures, cost effectiveness of test applied and difficult handling of larger population along with the constraint of test availability. In the present study competitive ELISA was used with high specificity (99.8%) and sensitivity (90.5%) for the detection of PPR virus antibodies in serum samples compared with gold standards like virus neutralization test VNT [12-14]. The implementation of cELISA aided in tracking the outbreaks of PPR disease in different geographical regions, measuring economic losses from the disease, epidemiology of the disease in different population of animals [1,15].

The mortality in PPR goats can be up to 100% in severe infections, but during milder outbreaks less than 50% mortality may be seen [16]. However, published information on the survivability of goats diagnosed with clinical PPR, under different antimicrobial therapies are sparse, if not absent. The results of the present study revealed that survivability in different treatment groups varies (Table 3). These treatment strategies are usually applied to combat PPR outbreaks in the field. Narayanan et al. [17] treated clinical cases of PPR were treated with broad-spectrum antibiotics like enrofloxcin, @5mg per kg body weight. Intestinal astringents like creta and kaolin were administered. Intravenous fluids like dextrose normal saline (10ml/kg body weight), was administered for the treatment of diarrhea and restoration of body fluid ionic balance for seven days as described by Wosu [18] and Abubakar and Irfan [19]. In contrast, we used above strategies along with use of PPR vaccine which proved the best in combating the outbreaks.

Virus induced immune-suppression attributable to leucopenia predisposes secondary bacterial infections, where bronchopneumonia is the most frequently observed bacterial complication in peste des petits ruminants virus (PPRV)-infected animals [16,20,21]. Common secondary infections include *Pasteurella* species [21]. The disease is highly endemic in South Asia, caused by lineage 4 of PPRV unlike Sub-Saharan countries where circulation of lineages 1-3 are reported [2]. Because the outcome of PPRV infections can be linked to secondary bacterial infections, it is important to treat them appropriately, subsequently an increased survivability might be achieved.

A possible explanation for outbreaks could be the excretion of PPR viral antigen in some body secretion. Abubakar et al. [22] explained the possible excretion of PPRV antigen in fecal material in which they described a possible mechanism of virus transmission following natural infection. This idea may demonstrate a potential method by which PPRV outbreaks occur spontaneously in areas not previously known to have circulating virus. Shedding of PPRV antigen in the fecal material of the recovered goats following a disease incursion put forward the possibility that goats may be shedding the PPR virus in their fecal matter. These findings are also in agreement with Ezeibe et al. [23] and may reinforce the idea that virus can sub-clinically infect animals and excrete and/or transmit virus to naïve ‘in contact’ animals.

## Conclusion

The main reason for these outbreaks could be the endemic nature of PPR disease in Pakistan and as there is no organized program for its vaccination. Therefore, it is recommended that proper importance should be given to treatment regimes as mentioned above as well as vaccination during the face of a PPR outbreak.
